# Genome-Wide Association Study of Yield Component Traits in Intermediate Wheatgrass and Implications in Genomic Selection and Breeding

**DOI:** 10.1534/g3.119.400073

**Published:** 2019-05-30

**Authors:** Prabin Bajgain, Xiaofei Zhang, James A. Anderson

**Affiliations:** * Department of Agronomy & Plant Genetics, University of Minnesota, St. Paul, MN and; † Department of Horticultural Science, North Carolina State University, Raleigh, NC

**Keywords:** QTL, GWAS, genomic selection, domestication, intermediate wheatgrass, Genomic Prediction, GenPred, Shared Data Resources

## Abstract

Intermediate wheatgrass (*Thinopyrum intermedium*, IWG) is a perennial grain crop with high biomass and grain yield, long seeds, and resistance to pests and diseases. It also reduces soil erosion, nitrate and mineral leaching into underground water tables, and sequesters carbon in its roots. The domestication timeline of IWG as a grain crop spans only 3 decades, hence it lags annual grain crops in yield and seed characteristics. One approach to improve its agronomic traits is by using molecular markers to uncover marker-trait associations. In this study, we performed association mapping on IWG breeding germplasm from the third recurrent selection cycle at the University of Minnesota. The IWG population was phenotyped in St Paul, MN in 2017 and 2018, and in Crookston, MN in 2018 for grain yield, seed length, width and weight, spike length and weight, and number of spikelets per spike. Strong positive correlations were observed among most trait pairs, with correlations as high as 0.76. Genotyping using high throughput sequencing identified 8,899 high-quality genome-wide SNPs which were combined with phenotypic data in association mapping to discover regions associated with the yield component traits. We detected 154 genetic loci associated with these traits of which 19 were shared between at least two traits. Prediction of breeding values using significant loci as fixed effects in genomic selection model improved predictive abilities by up to 14%. Genetic mapping of agronomic traits followed by using genomic selection to predict breeding values can assist breeders in selecting superior genotypes to accelerate IWG domestication.

Compared to annual crops, perennial crops provide better environmental services by reducing soil and water erosion and nutrient leaching as well as sequestering more carbon per square area (Tilman *et al.* 2006; Glover *et al.* 2015; Jungers *et al.* 2015). Perennials such as intermediate wheatgrass (*Thinopyrum intermedium*, IWG) add less stress to the environment from reduced chemical use and overall energy inputs while providing substantial agricultural value (Hoffman *et al.* 1995; Christian *et al.* 1997; Rogers *et al.* 2014). For example, IWG grains have been used to produce several food products and beverages (Wagoner 1990; Zhang *et al.* 2015; DeHaan and Ismail 2017). Because of its ecosystem services and food values, IWG has been identified as an ideal perennial crop for domestication (DeHaan *et al.* 2018). A cool-season perennial grass native to the Mediterranean and Eastern Europe (Tsvelev 1984), IWG is currently being improved for better agronomic qualities at the University of Minnesota in St Paul, MN, The Land Institute in Salina, KS, and University of Manitoba in Winnipeg, Canada.

Domestication of plant and animal species for human consumption has been under practice for several thousand years. The outcomes of this practice are numerous plant and animal species adapted to specific human needs and the environment of selection (Brown 2010; Fuller *et al.* 2010). Adaptations during the domestication process include fast changes in a suite of both physiological characteristics and genetic makeup, known as the ‘domestication syndrome’ (Hammer 1984). For example, the earliest traits selected in wheat during its domestication were reduced spikelet shattering, easier threshability, seed size, and other changes in plant morphology (Harlan *et al.* 1973). Selection of the best wheat plants adapted to these traits also improved other domestication syndrome traits such as reduced tiller number, straw strength, lodging resistance, and reduced seed dormancy (Dubcovsky and Dvorak 2007). Modern plant breeding has further improved these traits in virtually all domesticated crop species with the discovery and application of DNA markers.

Study and improvement of domestication traits have typically been done by using molecular markers in marker assisted selection assays and quantitative trait loci (QTL) mapping projects. Of different QTL mapping approaches, association mapping or genome-wide association study (GWAS) is one method where markers associated with the traits of interest are determined via linkage disequilibrium and allele frequency (Weir 2008). Such mapping studies uncovering the genetic factors controlling domestication traits have been carried out in multiple crop species including wheat, rice, maize, soybean, tomato, and common bean (Peleg *et al.* 2011). Many of these studies have shown that few gene clusters with large effects separate domesticated crops from their wild counterparts (Koinange *et al.* 1996; Gepts 2004). While some studies have indicated that multiple domestication-related traits are controlled by the same few genetic loci (Poncet *et al.* 2002; Cai and Morishima 2002), recent evidence suggests that a larger number of genes may also be involved. For example, Thomson *et al.* (2003) reported that 76 QTL control 13 traits in rice; nearly 1,800 candidate genes were found to be associated with domestication traits in maize (Hufford *et al.* 2012), and more than 500 loci were associated with 47 traits in foxtail millet (Jia *et al.* 2013). Of the traits studied, yield and yield component traits such as kernel size, seed dimensions, and spike characteristics are among the most prioritized traits because of their impact on agricultural production and food security.

During and post-domestication, higher yield has been a focus of breeding efforts in all plant species including cereal grasses. One of the very first traits to undergo domestication, resistance to shatter, is a key element in increasing yield by preventing grain loss during and after-harvest (Peleg *et al.* 2011). Other key components in maintaining high yields are spike-related morphological attributes such as length, weight, number of spikelets per spike as well as seed weight and seed dimensions (Slafer 2007; Qin *et al.* 2015). As yield is usually an amalgam of many subsidiary traits and has complex underlying genetic architecture, identification and selection of high-yielding genotypes requires the understanding of other single traits, mainly the yield component traits (Robson *et al.* 2013). A better understanding of genetic factors that control yield component traits will be important in improving IWG and establishing it as a successful perennial grain crop. One approach to accomplish this is through the discovery of genetic loci associated with these traits and their genetic variations in IWG breeding populations followed by their use in recurrent selection of superior genotypes. Genomic selection (GS) is one such tool that can be used to realize this goal. Using GS to improve IWG breeding populations has already been demonstrated as a sound strategy in accelerating its domestication (Zhang *et al.* 2016). Discovery of additional genomic regions linked with traits of interest will help understand their genetic control and further advance IWG’s domestication process.

The University of Minnesota started its IWG breeding program in 2011 and has since completed three breeding cycles. During these cycles, progress has been made in improving the germplasm for yield and yield component traits as well as other agronomic traits such as plant height and disease resistance. After statewide trials of candidate synthetic varieties during 2015-2018, the best candidate will be released in 2019 as the first synthetic IWG variety. Despite the progress made, IWG is still in the nascent stages of domestication. Some traits needing further improvement are low grain yield and small seed size compared to annual cereal crops. Determination of optimal plant height and biomass without sacrificing above- and below-ground plant performance is important as well. Synchronous flowering times and uniform maturity are also equally imperative in synthetic crossing blocks and cultivation of the variety.

With this in mind, this study was carried out with the following objectives: i) Discover markers and determine the genetic control of seed and yield component traits in the IWG breeding program at the University of Minnesota; ii) Characterize the amount of variability existent in the UMN IWG breeding germplasm by assessment of trait heritabilities, linkage disequilibrium, and population structuring; iii) Investigate the value of including significant markers detected by QTL mapping as fixed-effects in genomic selection models and their impact on trait predictive abilities.

## Materials and Methods

### Plant materials

The IWG population used in this study is from the third recurrent selection cycle (C3) at the University of Minnesota and is referred to as UMN_C3. It was initiated from 70 cycle 2 (UMN_C2) genets based on their genomic estimated breeding values (GEBVs) obtained from GS models trained on UMN_C2 agronomic data collected during 2014-2015. A genet is defined as a genetically unique organism and refers to individual plants in an outcrossing species such as IWG (Zhang *et al.* 2016). These genets were vernalized at 4° for 8 weeks during November-December 2015 and allowed to intercross in the greenhouse during January-March 2016. Eight random seeds were germinated in June 2016 from each mother plant, cloned into two groups in August 2016, and transplanted in the field as single replication in September 2016 in two MN locations: St Paul and Crookston. Transplanting was done with 1 m distance between the genets and plots were surrounded on all sides with IWG border plants. Plots were not fertilized in 2017, but 45 kg ha^-1^ of N was applied in April 2018 in St Paul and in May 2018 in Crookston. Weed control was primarily done with manual labor and mechanical cultivation. The herbicide Dual II Magnum (S-Metolachlor 82.4%, Syngenta) was applied in April of both years at a rate of 1.2 L per ha. Post-harvest, plants were mowed to a height of 15-20 cm. The environments St Paul 2017, St Paul 2018, and Crookston 2018 are referred to as StP17, StP18, and Crk18, respectively. The Crk17 environment was abandoned due to flooding of the field that resulted in poor plant establishment. Because of plant death and loss of genets between two locations, 451 genets were used in the final association analysis.

### Genotyping

From each genet, 10-15 cm of leaf tissue was collected and dried on silica for 5 d. DNA was extracted from ground leaf tissue using the BioSprint 96 DNA Plant Kit (QIAGEN, Valencia, CA). Extracted DNA was digested with *Pst*I and *Msp*I to create double digested libraries and sequenced in 192-plexed libraries on Illumina’s Hiseq 2500. Obtained sequences were passed through a quality filter of *Q* > 30 then de-multiplexed to obtain reads for each individual genet. Reads were aligned to the draft IWG reference genome v2.1 (Thinopyrum intermedium Genome Sequencing Consortium) using bwa (Li and Durbin 2009), and samtools+bcftools (Li 2011) for SNP calling. SNPs with minor allele frequency (MAF) of less than 5% and more than 20% missing data were removed. The resulting dataset of 8,899 SNPs were imputed using the LD-kNNi method (Money *et al.* 2015) in Tassel version 5.2.41 (Bradbury *et al.* 2007) using 30 nearest neighbors. Imputation accuracy was calculated within Tassel by randomly masking known genotypes of 20–50% alleles in the input file before imputation and comparing with allelic predictions of masked genotypes. Forced-imputation was not carried out if the missing genotype of a locus could not be resolved.

### Phenotyping and statistical analysis

The UMN_C3 IWG genets were phenotyped in 2017 and 2018 in St. Paul, MN and in 2018 in Crookston, MN. The panel was evaluated for multiple agronomic traits, of which we focus our GWAS analysis on seven yield-related traits: 1) grain yield, 2) thousand kernel weight (TKW), 3) seed length, 4) seed width, 5) number of spikelets per spike, 6) spike weight, 7) and spike length. These traits were measured by harvesting 10 mature spikes per plant and drying them at 32° for 72 h. Spikes were first measured for weight, length, and spikelet count followed by mechanical threshing to obtain 10-spike yield. All remaining spikes from each genet were also harvested, dried, threshed using Wintersteiger LD 350 (Wintersteiger Inc, Salt Lake City, USA), and combined with 10-spike yield to obtain total plant yield. Approximately 100-300 de-hulled seeds from each genet were scanned using Marvin seed analyzer (GTA Sensorik GmbH, Germany) to obtain seed length and width. Imaged seeds were weighed to obtain TKW.

Trait data were passed through a mixed model equation to correct for environmental variability (*i.e.*, the trial effect) and obtain the best linear unbiased estimation (BLUE) of each genet using the MIXED procedure in SAS (v.9.3.1; Sallam *et al.* 2015). The fixed effect estimate obtained for a particular environment was removed from the trait value for each genet in that environment to obtain adjusted BLUE values and used in association analysis. Broad-sense heritability (H) of the traits were calculated on a genet mean basis using the formula:

$$\text{H}=\sigma_{\text{g}}^{2}/\left(\sigma_{\text{g}}^{2}+\sigma_{\text{e}}^{2}/\text{η}\right)$$

where:

σ_g_^2^ is the genetic variance,

σ_e_^2^ is the error variance that includes the genotype × environment effect and residuals, and

η is the number of years.

### Linkage disequilibrium & population structure

Linkage disequilibrium (LD) among the genome-wide markers was calculated using Tassel version 5.2.41 with sliding window size of 1000 markers. Obtained r^2^ values were plotted against both physical and genetic distances with a LOESS curve fitted to display LD decay. LD decay distance was estimated using the method of Hill and Weir (1988) and assessed at the conventionally accepted *r^2^* value of 0.2 (Vos *et al.* 2017). Genetic distances between the SNPs were assigned from highly similar SNP sequences aligned with the sequences reported in the IWG consensus map (Kantarski *et al.* 2017). The command *magicblast* in ncbi-magicblast-1.3.0 was used after converting the consensus sequences into a local database using *makeblastdb* (Boratyn *et al.* 2018). BLAST output was parsed to retain alignments with e-value of 1E-10 with 90% sequence similarity and minimum alignment length of 25 base pairs.

The same 8,899 SNPs were used in STRUCTURE (Pritchard *et al.* 2000) with subgroups K = 1 to 10 used to determine the optimal number of population subgroups. Using the admixture model with STRUCTURE, K = 1 through 10 were tested with 100,000 reps with the first 25,000 declared as burn-ins with 10 replicates for each value of K. The outputted K statistics were analyzed using Structure Harvester (Earl and vonHoldt 2012) to determine the optimal K number. Results from Structure Harvester suggested K= 2 as the most likely scenario for UMN_C3, yet moderately strong signal was also observed at K = 6. Therefore, a network-distance based clustering of the genotypes was carried out in NetStruct to confirm the number of sub-populations (Greenbaum *et al.* 2016). Threshold values of 0.01 to 0.20 were tested at increments of 0.05 using the spectral analysis algorithm (Csardi and Nepusz 2006). Strength of association distribution analysis was carried out on optimal community values and plotted over principal component (PC) values calculated using the function *prcomp* in R.

### Association analysis & genomic selection

The program Genome Association and Prediction Integrated Tool (GAPIT; Lipka *et al.* 2012) was used for association analysis. In GAPIT, the uncompressed mixed linear model (MLM) was used with the Q matrix obtained from STRUCTURE at K = 2 as covariates. PC values were not used as covariates as model optimization with up to 10 PC values showed no improvement. Significant QTL were declared at *P* < 0.001 because Bonferroni corrected p-values were found to be restrictively conservative. For all significant markers, the percentage of explained phenotypic variation (*R*^2^), major and minor allele frequencies, and allelic effects are reported.

SNP markers significantly associated with the traits were used as fixed effects in genomic selection models to study how they affected predictive abilities of each trait. This was carried out in rrBLUP (Endelman 2011) using fourfold cross validation where 75% of the UMN_C3 panel was used as the training population and the remaining 25% as the validation set. Four scenarios were evaluated: 1) no markers declared as fixed effects, 2) top 10 loci for each trait (SNPs with the best 10 *R*^2^ values) as fixed effects for the specific trait, 3) all significant markers for each trait as fixed effect for that specific trait, and 4) all significant SNPs detected for all traits as fixed effects for each trait. Each scenario was run for 100 replications and correlations between GEBVs of the masked validation set and the training population were averaged.

### Data availability

All supplemental materials are available at Figshare including phenotype (File S1) and genotype data (File S2). Sequences of entire UMN_C3 population have been uploaded to NCBI’s sequence read archive under BioProject PRJNA518132. Other data and germplasm associated with the UMN_C3 intermediate wheatgrass population are available upon request. Supplemental material available at FigShare: https://doi.org/10.25387/g3.7701509.

## Results

### SNP discovery, population structure, & linkage disequilibrium

Reference-based read alignment followed by SNP calling led to discovery of 3,291,243 SNPs in the UMN_C3 breeding population of which 1,651,365 remained after discarding those with MAF lower than 5%. Removal of SNPs with proportion of missing alleles > 20% reduced this number to 8,899 with an average of 424 SNPs per chromosome (Table S1). Imputation of missing alleles using LD-kNNi method in Tassel version 5.2.41 using 30 nearest SNPs lowered the overall missing allele proportion from 20 to 1.8% with an imputation accuracy of 94.6%.

**Figure 1:**
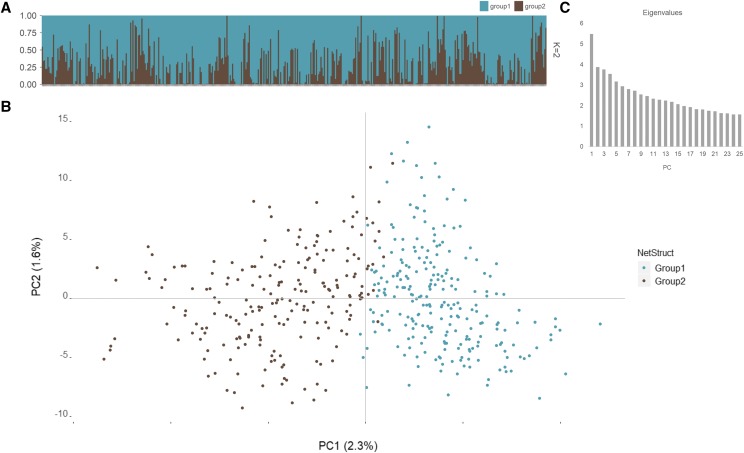
Population structure inferred by STRUCTURE and NetStruct. For both methods, K = 2 are plotted, along with the first 25 eigenvalues.

Estimation of population structure was first done with STRUCTURE, which implements a Bayesian clustering method. Log likelihood values were analyzed using the Evanno method in Structure Harvester to determine an optimal K value. The maximum ΔK was observed at K = 2 with a second moderately high ΔK value at K = 6 (Table S2). Membership (genets assigned) proportions in the two clusters when K = 2 are 72% and 28% (Figure 1A). Because of a very narrow origin of current IWG breeding germplasm, higher values of K are not expected. Therefore, an additional method of population clustering based on network analysis was implemented in NetStruct. Evaluation of threshold values from 0.010 to 0.024 resulted in division of UMN_C3 into two clusters (communities, in NetStruct terminology) whereas values of ≥ 0.030 split the genets into ≥ 368 clusters, which is erroneous. Hence, for the purpose of this study, K = 2 was determined as the best estimation of population structuring for UMN_C3. Strength of association distribution analysis in NetStruct of the two clusters showed a difference of only 7.59E-06, establishing that the proposed two clusters are very closely related with each other (Figure 1B). Distribution of the first 25 eigenvalues is shown in Figure 1C, from which the amount of genetic variation explained by the first two PC axes were calculated at 2.3% and 1.6%, respectively.

**Figure 2:**
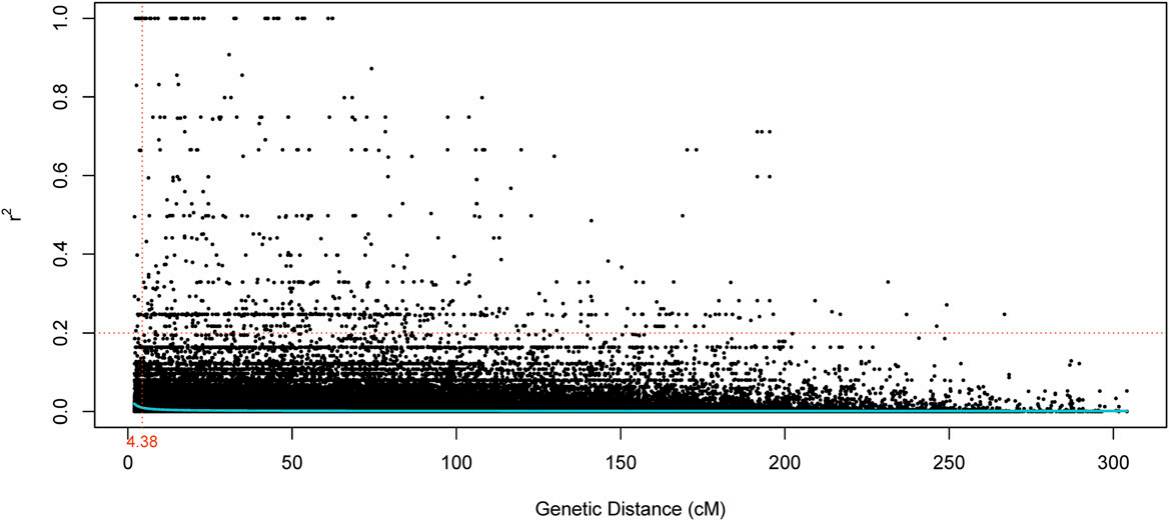
Linkage disequilibrium (r^2^) plotted against the genetic distance (cM) in UMN_C3 population. Blue line is fitted to display the distribution.

Half decay distance in the UMN_C3 population at arbitrary nominal level of r^2^ = 0.20 was found to be 4.38 cM, according to Hill and Weir (1988) method (Figure 2). In terms of physical distance, this distance was 0.7 mega base pairs (Mbp).

### Phenotypic data variation & heritability

**Figure 3:**
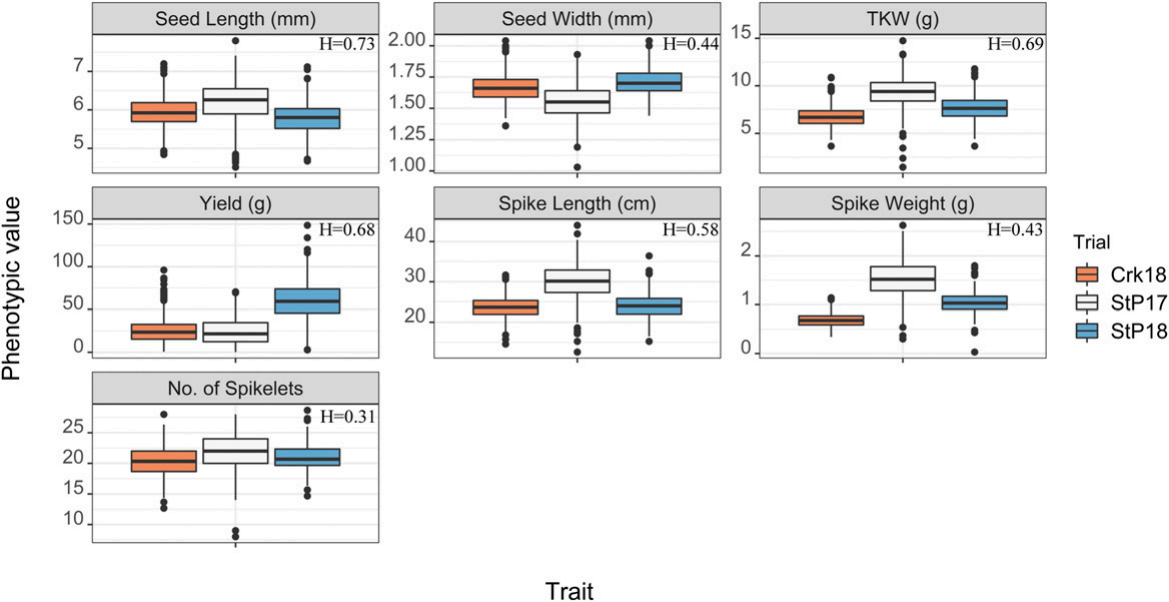
Boxplots of phenotypic data collected on UMN_C3 in St Paul in 2017 and 2018, and in Crookston, MN in 2018. For each trait, its broad sense heritability (H) is displayed on top right of each plot.

Environment StP17 – the first year of UMN_C3 – had the largest mean values for spike and seed related traits whereas StP18 had higher total grain yield (Table 1, Figure 3). Overall, the highest single plant yields observed were 70.5 g, 148.5 g, and 96.0 g in StP17, StP18, and Crk18 respectively. While no single genet was the best overall in all three environments for plant yield, nine out of top 25 highest-yielding genets were shared in at least two environments. Average seed weight, measured in terms of thousand kernel weight (TKW), were 14.7 g, 13.0 g, and 10.9 g in StP17, StP18, and Crk18 respectively. The longest as well as shortest seeds were observed in StP17 at 7.8 mm and 4.5 mm, respectively. StP18 had the widest seeds at 2.2 mm, relative to that of 1.9 mm and 2.0 mm in StP17 and Crk18, respectively.

**Table 1:** Distribution of phenotypic values from StP17, StP18, and Crk18 in the UMN_C3 IWG breeding population

Trait	Environment	range	mean ± SD
**Seed Length (mm)**	**StP17**	4.5 - 7.8	6.2 ± 0.5
	**StP18**	4.7 - 7.1	5.8 ± 0.4
	**Crk18**	4.8 - 7.5	6.0 ± 0.4
**Seed Width (mm)**	**StP17**	1.0 - 1.9	1.6 ± 0.1
	**StP18**	1.4 - 2.2	1.7 ± 0.1
	**Crk18**	1.4 - 2.0	1.7 ± 0.1
**TKW (g)**	**StP17**	1.4 - 14.7	9.3 ± 1.5
	**StP18**	2.4 - 13.0	7.6 ± 1.3
	**Crk18**	3.7 - 10.9	6.8 ± 1.1
**Yield (g)**	**StP17**	0.1 - 70.5	23.1 ± 14.6
	**StP18**	1.7 - 148.5	57.7 ± 23.9
	**Crk18**	0.6 - 96.0	24.4 ± 14.6
**Spike Weight (g)**	**StP17**	0.3 - 2.6	1.5 ± 0.4
	**StP18**	0.0 - 1.9	1.0 ± 0.2
	**Crk18**	0.3 - 1.1	0.7 ± 0.1
**Spike Length (cm)**	**StP17**	12.6 - 44.0	29.9 ± 4.1
	**StP18**	15.2 - 36.4	24.0 ± 2.9
	**Crk18**	14.6 - 31.7	23.8 ± 2.6
**No. of Spikelets**	**StP17**	8.0 - 28.0	21.9 ± 2.7
	**StP18**	12.0 - 89.3	21.1 ± 5.3
	**Crk18**	12.7 - 89.7	20.7 ± 4.9

**Figure 4:**
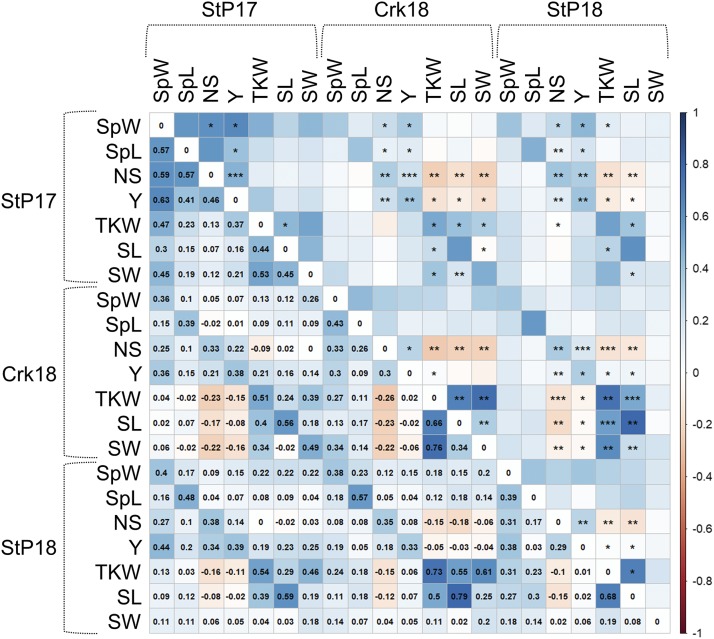
Heat-map of coefficient of correlations among the traits collected for UMN_C3 in StP17, StP18, and Crk18. Lower triangle contains the *r* values and the symbols *, **, and *** in the upper triangle denote significance at p values of 0.05, 0.01, and 0.001, respectively. SL: seed length; SW: seed width; Y: grain yield; TKW: thousand kernel weight; SpL: spike length; NS: no. of spikelets per spike; SpW: spike weight.

In all environments, strong positive correlations were observed among yield component traits such as spike weight, spike length, number of spikelets, TKW, and grain yield. The highest trait correlations were observed between seed lengths in StP18 and Crk18 with the coefficient of correlation, *r* = 0.79 followed by TKW and seed width in Crk18 (*r* = 0.76) and TKW in StP18 and TKW in Crk18 (*r* = 0.73) (Figure 4). Correlations between seed length and width in StP17 and Crk18 were significant (*r* = 0.45 and 0.34, respectively) but was poorly correlated in the second year trial in St Paul (*r* = 0.1). Few negative yet significant correlations were also present between several trait pairs within and across the environments. The lowest correlations observed in the dataset were between no. of spikelets and TKW in Crk18 (*r* = -0.26). In all environments, low correlations were observed between no. of spikelets and TKW (*r* = -0.26 to 0.13), and seed length and seed width (*r* = -0.02 to 0.45). Broad-sense heritability estimates were medium to large with the highest values observed for yield (0.68), TKW (0.69), and seed length (0.73) (Figure 3).

### Significant SNP markers & favorable alleles

**Figure 5:**
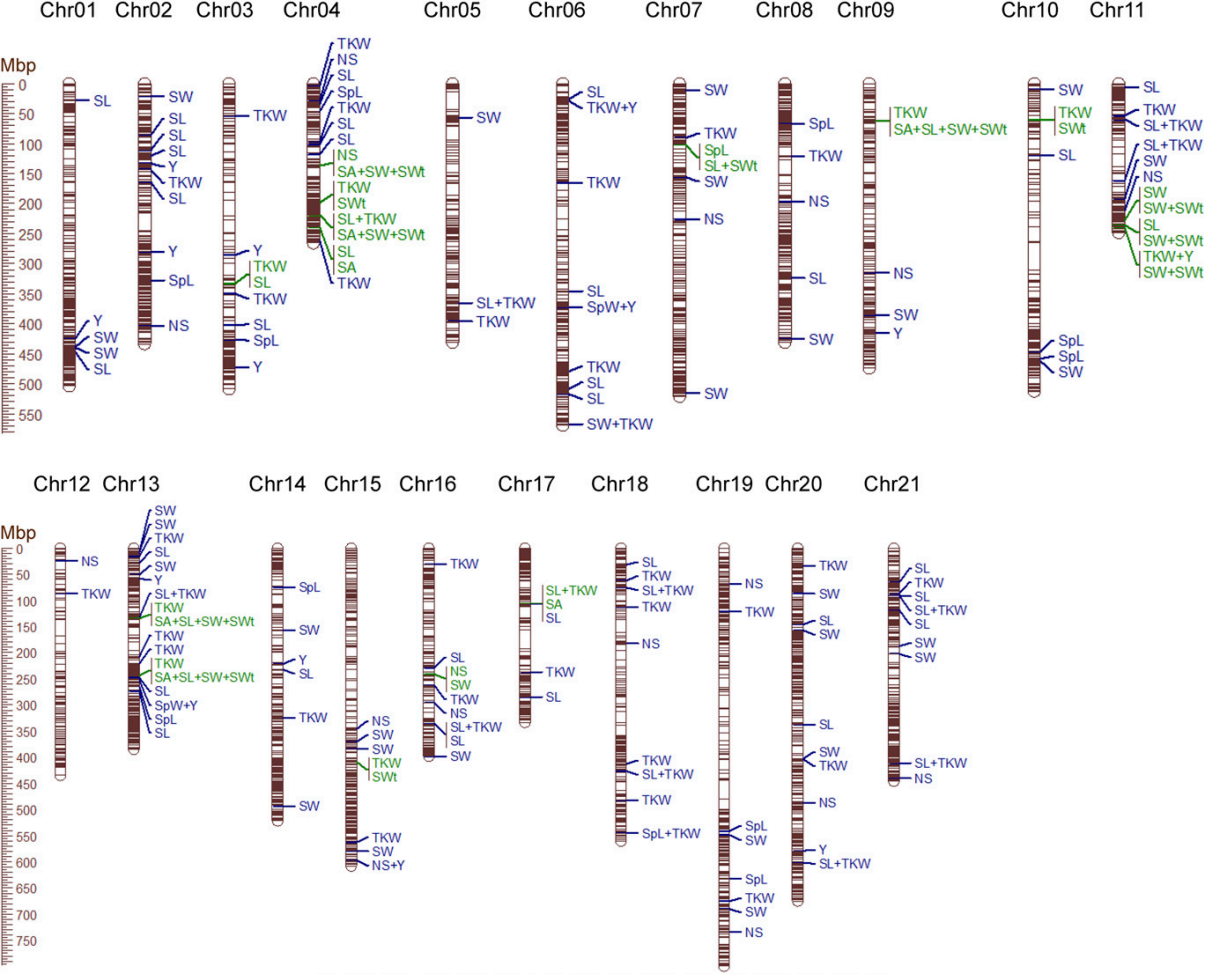
QTL associated with seven agronomic traits in UMN_C3 IWG breeding population. Blue colored loci indicate QTL detected in this study. Green colored loci indicate QTL detected by Zhang *et al.* (2017) and this study that are located within 5 Mbp of each other. SL: seed length; SA: seed area; SWt: seed weight; SW: seed width; Y: grain yield; TKW: thousand kernel weight; SpL: spike length; NS: no. of spikelets per spike; SpW: spike weight.

Fitting the MLM in GAPIT using the Q matrix obtained from STRUCTURE led to detection of 154 loci in all 21 chromosomes that were significantly associated with the 7 traits (Figure 5, Table S3). The largest number of QTL were detected for TKW with 53 total in all chromosomes except Chromosome 1. The fewest QTL (3) were discovered for spike weight in Chromosomes 4, 6, and 13 with the percent of phenotypic variance explained (*R*^2^) values of 2.6–4.1%. The highest *R*^2^ values in the dataset were observed for seed width (10.8%) which was associated with 30 loci in 16 chromosomes. Forty-five QTL were detected for seed length in 16 chromosomes with *R*^2^ values of 2.3–4.9%. Eleven and 17 QTL were located in 9 and 13 chromosomes for traits spike length and number of spikelets, respectively. For grain yield, 12 small effect QTL ranging in *R*^2^ values from 2.5–3.8% were detected in eight chromosomes. Most QTL (15) were found in Chromosome 13 and the fewest (2) were found in Chromosome 12. The number of QTL detected was strongly correlated with number of SNPs per chromosome (*r* = 0.54) but was not correlated with chromosome lengths (*r* = -0.07).

Nineteen QTL were shared among the traits and are summarized in Table 2. Twelve QTL were common between TKW and seed length; two between spike weight and yield, and TKW and yield; and one each between TKW and seed width, TKW and spike length, and number of spikelets per spike and yield. Forty QTL were detected in at least two of the three environments. Of these, five were detected in all three environments: four for seed length and one for TKW. Of the QTL observed in only two out of three environments, 17 were for TKW, 15 for seed length, two for seed width, and one each for grain yield, number of spikelets, and spike length.

**Table 2:** Significant QTL common between at least two traits

						Seed Length		Seed Width		TKW		Yield		Spike Length		Spike Weight		No. of Spikelets	
SNP	Chrom	Pos (Mbp)	Major Allele	Minor Allele	MAF	-log(p)	R^2^	-log(p)	R^2^	-log(p)	R^2^	-log(p)	R^2^	-log(p)	R^2^	-log(p)	R^2^	-log(p)	R^2^
**S04_220130053**	4	220.13	G	A	0.20	3.38	2.59	—	—	3.35	2.40	—	—	—	—	—	—	—	—
**S05_366528709**	5	366.53	G	C	0.42	5.22	4.30	—	—	3.35	2.40	—	—	—	—	—	—	—	—
**S06_30049280**	6	30.05	T	C	0.19	—	—	—	—	3.14	2.22	3.08	2.57	—	—	—	—	—	—
**S06_373070997**	6	373.07	A	C	0.22	—	—	—	—	—	—	3.32	2.77	—	—	3.14	2.58	—	—
**S06_568793265**	6	568.79	G	A	0.45	—	—	4.90	3.95	4.43	3.33	—	—		—	—	—	—	—
**S11_161626009**	11	161.63	C	T	0.16	3.11	2.35	—	—	3.65	2.66	—	—	—	—	—	—	—	—
**S11_241092955**	11	241.09	G	A	0.47	—	—	—	—	3.74	2.73	3.30	3.78	—	—	—	—	—	—
**S11_59775443**	11	59.78	G	A	0.26	3.42	2.63	—	—	3.57	2.59	—	—	—	—	—	—	—	—
**S13_134782913**	13	134.78	A	G	0.10	3.27	2.49	—	—	3.81	2.79	—	—	—	—	—	—	—	—
**S13_252462690**	13	252.46	T	G	0.34	—	—	—	—	—	—	4.06	3.51	—	—	4.70	4.14	—	—
**S15_600918584**	15	600.92	C	G	0.41	—	—	—	—	—	—	3.32	2.80	—	—	—	—	4.55	3.87
**S16_336657077**	16	336.66	C	T	0.42	3.22	2.45	—	—	3.16	2.23	—	—	—	—	—	—	—	—
**S17_108653775**	17	108.65	A	T	0.18	3.70	2.89	—	—	3.29	2.35	—	—	—	—	—	—	—	—
**S18_426508862**	18	426.51	A	G	0.08	3.52	2.72	—	—	3.06	2.16	—	—	—	—	—	—	—	—
**S18_545444320**	18	545.44	T	A	0.05	—	—	—	—	3.01	2.03	—	—	3.35	2.75	—	—	—	—
**S18_75576716**	18	75.58	C	G	0.18	3.36	2.58	—	—	4.06	3.01	—	—	—	—	—	—	—	—
**S20_603356555**	20	603.36	G	C	0.21	4.51	3.64	—	—	3.25	2.31	—	—	—	—	—	—	—	—
**S21_411630228**	21	411.63	C	T	0.13	3.43	2.64	—	—	3.67	2.67	—	—	—	—	—	—	—	—
**S21_90762051**	21	90.76	A	G	0.13	5.85	4.91	—	—	3.65	2.66	—	—	—	—	—	—	—	—
**Total R^2^**							36.18		3.95		40.53		15.42		2.75		6.72		3.87

In this study, favorable alleles are defined as those that are significantly associated with the QTL and have positive allelic effect estimates. Of the 1,078 significant alleles (154 loci × 7 traits), 55% had major alleles as favorable and 45% had minor alleles as favorable (Table S4). Only six of the 154 significant loci had all major alleles as favorable for all seven traits, and only five loci had only minor alleles that were favorable for all traits. Between the two allele groups, *i.e.*, group of favorable major alleles *vs.* group of favorable minor alleles, no significant differences were observed in allelic effect estimates or *R*^2^ values (*t*-test *p-value* > 0.1). The highest proportion of favorable alleles at QTL for each trait was observed for TKW and seed length (27% each) whereas spike weight had the least (0.3%), likely a function of number of QTL detected for these traits.

### Genomic prediction using significant markers

**Figure 6:**
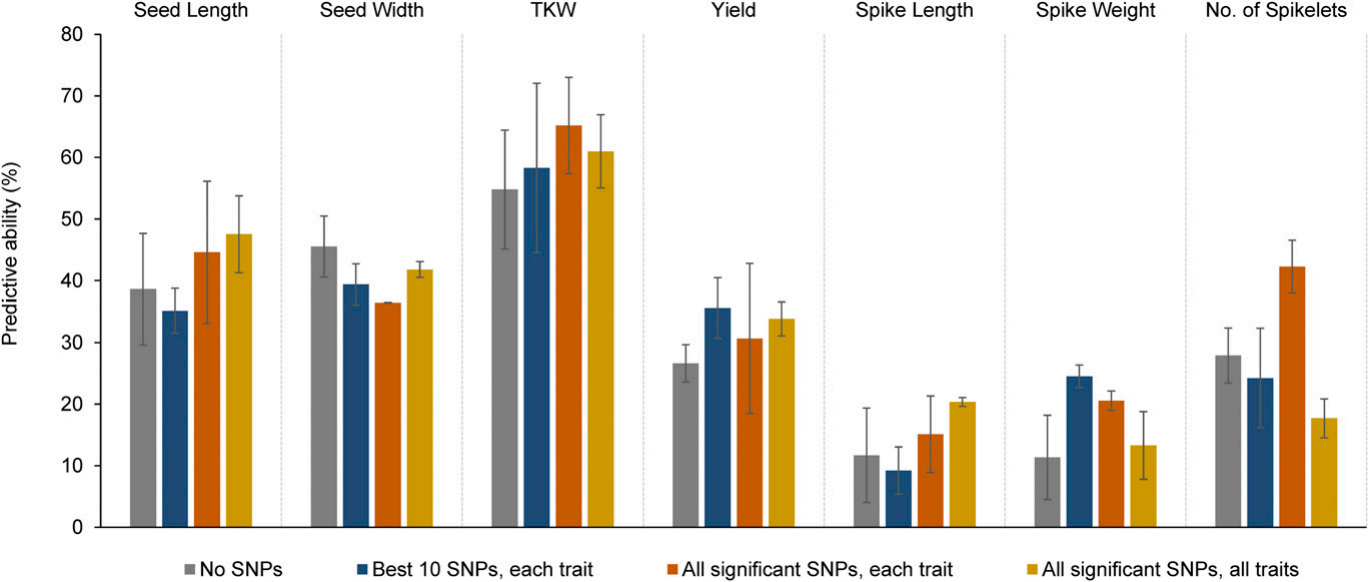
Effect on predictive abilities of traits when using significant SNPs as fixed effects in genomic selection models. Best 10 SNPs for each trait are the ones with highest amount of percentage of phenotypic variance explained (R^2^). Error bars represent the standard deviation of predictive ability values obtained from each model.

Using significant SNP markers from GWAS as fixed effects in genomic selection models improved the predictive ability of all traits except seed width (Figure 6). Relative to the predictive abilities obtained when no SNPs were used as fixed effects, increases of 2–14% were observed when using significant SNPs as fixed effects. Of the four scenarios that were implemented, no single scenario was the best overall for all traits. Providing SNPs as fixed effects made the prediction models perform worse in a few cases, and appeared to be dependent on the trait as well as the no. of SNPs used as fixed effects. The most interesting as well as contrasting observations were for no. of spikelets: using SNPs only significant for the trait increased predictive ability by 14% (the best increase %) whereas using SNPs significant for all traits resulted in 10.2% reduction in predictive ability, the highest reduction in predictive ability.

## Discussion

Annual crops such as wheat, barley, maize, and sorghum have benefitted from long selection histories with domestication commencing approximately 10,000 years ago (Dillon *et al.* 2007; Meyer *et al.* 2012). Because of the impact these crops have made for several thousand years on human lives, they are widely cultivated and are ingrained into many cultures and countries. On the other hand, novel crops such as IWG with very short domestication histories have many traits that need to be improved simultaneously in order to establish themselves as successful crops. Improving domestication-related traits and several other agronomic traits by uncovering genomic loci controlling the traits and accumulating favorable alleles in a breeding germplasm is necessary to expedite the domestication timeline of these new crops.

The University of Minnesota started breeding and improving IWG as a grain crop in August 2011. We have recently completed the third cycle of selection (UMN_C3) and initiated the fourth cycle in August 2018. The UMN_C3 population discussed in this study was phenotyped at two MN locations: St Paul and Crookston over two years, 2017-2018. Multiple trait pairs exhibited strong correlations within and across the environments, especially seed length (the correlation coefficient ‘*r*’ ranged 0.56-0.79), spike length (0.39-0.57) and grain yield (0.33-0.44). We also observed weak to moderately strong and significant negative correlation between trait pairs, yet these negative associations were mostly among traits in different environments, and thus may not have meaningful implications. Of the few within-environment negative correlations, the most notable ones were no. of spikelets with TKW and no. of spikelets with seed length in both locations for the year 2018; for St Paul 2017, these correlations were barely positive. Negative correlation between no. of spikelets and TKW are not uncommon in wheat (Deng *et al.* 2017; Philipp *et al.* 2018), but the negative relationship between no. of spikelets and seed length is concerning because we desire high values for both traits.

Population structure can increase discovery rates of false SNP-trait associations if unaccounted for (Lander and Schork 1994; Yu *et al.* 2005). We therefore investigated the level of population structure present in UMN_C3 population prior to running GWAS analysis. In their study, Zhang *et al.* (2017) reported high probabilities for K = 2 and 3 in the UMN_C1 population. Our analysis also determined K = 2 as the most probable solution followed by K = 6 but K = 3 was non-existent. This could be due to UMN_C3 1) being genetically different from UMN_C1, and 2) not adhering to assumptions made by the program STRUCTURE. STRUCTURE assumes that all K groups are equidistantly located and tends to lose efficiency when grouping individuals into smaller clusters of related populations (Kalinowski 2011). As the first three principal component values explained only 5.4% of the total genetic variation (< 14% from the first 10 axes), a low level of differentiation among the UMN_C3 genets can be expected. This is not surprising since the origin of UMN IWG breeding germplasm can be traced back to just 66 half-sib families (Zhang *et al.* 2016). A second program (NetStruct) that implements network-based clustering was used to group the UMN_C3 genets also suggested K = 2. Because of these results, the Q matrix obtained from STRUCTURE with K = 2 was used as a covariate in GWAS despite the low level of population structure.

Decay of LD in UMN_C3 was at 0.7 Mbp or 4.38 cM when *r*^2^ = 0.2. The LD decay in UMN_C1 was estimated to be 5 cM when *r*^2^ = 0.2 (Zhang *et al.* 2016) and 2 cM when *r*^2^ = 0.16 (Zhang *et al.* 2017). Compared to both studies by Zhang *et al.* our population has more SNP markers and used the IWG v2.1 reference genome to call SNPs. As our marker set offers a higher quality and better genomic resolution, we are confident that our estimation of LD is realistic. Despite the differences in LD values, all studies confirm a rapid decline in LD within a short physical distance. This is typical of outcrossing plant species as they tend to have high rates of effective recombination (Wright *et al.* 2008). Decay of LD within short distances is considered to offer more precise mapping of causative genetic variants (Gaut and Long 2003). Accurate QTL mapping is helpful in candidate gene discovery and in identifying tightly linked diagnostic markers that can be used in marker-assisted selection.

GWAS of seven yield component traits in the UMN_C3 IWG breeding population led to detection of 154 genetic loci associated with the traits. Nineteen QTL were shared among multiple traits. On one hand, the discovery of common QTL is not only an indication of a robust QTL mapping approach, but it also indicates that multiple traits can be improved simultaneously. On the other hand, obtaining same QTL for multiple traits or more shared QTL among several traits is also difficult due to several limitations such as genetic differentiation in a population, environmental effects, and residual error. In our analysis, nearly all significant loci explained small proportions of the observed phenotypic distribution, except for seed weight, where few loci had *R*^2^ values > 5% with the highest being 11%. IWG has small seeds relative to wheat: the median seed width and seed weight of IWG are 53% and 74% less (Zhang *et al.* 2017). Hence, detection of loci with large effects is vital, especially for seed size and weight, to increase trait values and attain larger seeds and higher yield of IWG. Overall, the most significant loci had small *R*^2^ values. This suggests that selection of genotypes based on *per se* phenotypic performance to obtain superior progeny might be an arduous task. This is because several rounds of phenotypic selection in multiple environments are needed to increase the frequencies of favorable alleles and fix them in the breeding population. In fact, we studied how the frequencies of favorable alleles of the 154 significant loci detected in UMN_C3 had changed compared to UMN_C1. We found 99 common loci (out of 154) between UMN_C1 and UMN_C3 of which 70 (71%) had higher allele frequencies in UMN_C3 relative to that in UMN_C1 (Table S5). However, this increase in favorable allele frequencies was not significant (*t*-test *P* value of 0.06 at *α* = 0.05). Nonetheless, nearly three-fourths of the significant loci detected in UMN_C3 population have higher favorable allele frequencies compared to UMN_C1, suggestive of strong selective pressure directing the advancement of several agronomic traits. We expect this trend to continue in our future IWG breeding populations as we emphasize the improvement of yield and yield-component traits.

In an attempt to compare QTL detected by Zhang *et al.* (2017) and this study, sequences of significant SNP markers from their study were aligned with sequences of significant SNP markers from our study. This produced zero matches; hence an alternative approach was used wherein sequences from their study were BLAST-searched against the IWG v2.1 genome to obtain SNP positions. Position of SNPs were extracted and investigated if they fell within 5 Mbp up or downstream of SNP markers significant in this study. This led to detection of 24 QTL in 13 chromosomes from the study of Zhang *et al.* (2017) within 5 Mbp of 19 QTL in our study (Figure 5, Table S6). Eight of 24 QTL were less than 1.5 Mbp away from our significant loci, and could be the same QTL. Additionally, 74% of similar QTL between the two studies were associated with yield component traits such as seed length, width, area, and TKW. Other similar QTL were mapped for different traits, *e.g.*, four QTL found by Zhang *et al.* (2017) for seed width were mapped for no. of spikelets in our study, and one QTL each for seed length and TKW detected by Zhang *et al.* (2017) were associated with spike length in this study.

Complex traits such as yield are usually controlled by many genetic factors with small effects (Quarrie *et al.* 2006; Bernardo 2008). This reduces the efficacy of marker-assisted selection because individual QTL effects are small, poorly estimated, and may change based on genetic background and environment. Likewise, increasing genetic gain for a complex trait over time from phenotypic selection only can be challenging because of the time and effort required to accumulate multiple small effect loci. This problem is compounded in the case of perennial species like IWG where phenotyping methods and management practices are labor and resource intensive due to a long life cycle of the plant. In this scenario, genomic selection (GS) can be a sound supplementary selection approach to improve multiple traits because it analyzes the effect of genome-wide loci on many traits instead of focusing on a few genes controlling a specific trait (Jannink *et al.* 2010). GS is able to report the overall genetic variance by evaluating the effects of all genome-wide markers on a given population and thus, the marker effects can be combined to predict the breeding performance of an individual (Meuwissen *et al.* 2001). Its applicability in obtaining a genome-wide summary of loci involved with polygenic traits instead of focusing on a few traits controlled by few large-effect markers has practical implications for improving IWG. For IWG and other novel crops that are in the early stages of domestication from their wild states, it is also important to improve several agronomic traits together. These traits include domestication-related traits such as non-shatter, free threshing, seed fertility as well as important agronomic traits such as yield, lodging, height, disease resistance, and seed quality traits. Use of GS can significantly improve these traits by relying on only a fraction of resources that would otherwise be needed with phenotypic selection alone (Zhang *et al.* 2016).

In our IWG breeding program, phenotypic data from year 1 are used to train GS models. The best model is then used to predict the performance of several thousand breeding genets from which the best ones are selected and intercrossed to obtain progeny for the next breeding cycle. Using SNP markers significantly associated with our traits increased trait predictions made in GS models by up to 14%. This was expected as it is known in both theory (Bernardo 2014) and from empirical data in different crop species that prediction accuracy increases when major genes and QTL are fitted as fixed effects in GS models (Spindel *et al.* 2015; Sarinelli *et al.* 2019). Therefore, routine application of GWAS and using significant loci as fixed effects will remain an indispensable strategy for improvement of UMN IWG germplasm. As a follow-up to this study, we plan to use SNP markers linked with traits in previous IWG QTL mapping studies to determine if they further improve GS predictions. We are also investigating the use of haplotype blocks, after the incorporation of dominance and epistatic effects in GS models, to increase predictive abilities. If proven successful, the new models will be implemented regularly in our GS-based breeding to improve IWG.

## Conclusions

In this study, we presented and discussed the results from GWAS of seven yield component traits in intermediate wheatgrass, a new perennial grain crop undergoing domestication. Observed strong correlations among yield component traits imply that improvement of correlated traits can be expected when selection pressure is applied on other traits. The UMN IWG breeding program implements genomic selection for trait improvement, which has increased the frequencies of most favorable alleles associated with agronomic traits, as observed in the most recent selection cycle. Using significant markers detected by GWAS in genomic selection models improved trait predictive abilities. Considering that the perenniality of IWG makes phenotyping more challenging and resource intensive, discovery of key QTL enables breeders and geneticists to make steady improvement of important agronomic traits and establish IWG as a successful crop with a positive impact on agricultural sustainability and food security. We expect these results to be applicable and contributive in domestication and improvement efforts of other novel annual and perennial plant species.
